# The outcomes of a healing environment and clustering nursing care on premature infants' vital signs, pain, and sleeping

**DOI:** 10.25122/jml-2022-0253

**Published:** 2022-11

**Authors:** Abdelaziz Hendy, Sabah Saad Alsharkawy, Nahed Saied El-Nagger

**Affiliations:** 1 Pediatric Nursing Department, Faculty of Nursing, Ain Shams University, Cairo, Egypt; 2 Faculty of Nursing, October University, Giza, Egypt

**Keywords:** clustering nursing care, healing environment, pain, premature infant, sleep vital signs

## Abstract

Our study aimed to assess the effects of creating a healing environment and clustering nursing care on premature infants' vital signs, pain, and sleeping. The study had an experimental research design for the control and study group, each with 53 premature infants. We collected the data through the Vital Signs Sheet, Premature Infant Pain Profile, and Neonatal behavioral state. We used T-tests and chi-square tests to assess the differences between groups. There was a highly statistically significant difference between the study and control groups concerning respiration (p-value<0.01) and heart rate, systolic blood pressure, and O^2^ saturation (p-value<0.05). 90.6% of participants in the study group had a mild total premature infant pain profile, while 37.7% of the control group had a moderate total premature infant pain profile score. Applying a healing environment and clustering nursing care significantly improved respiration, heart rate, oxygen saturation, and systolic blood pressure. Furthermore, it increased sleep time and decreased wake state and pain score.

## INTRODUCTION

Worldwide, premature newborns are the group that is most at risk. Preterm births are thought to affect 15 million babies yearly, and the number is growing. One of the most common problems with preterm admission to a neonatal unit is stress, which can have immediate and long-term implications on preterm health, development, and growth. The neonatal team can minimize the amount of stress in preterm infants by monitoring stress levels and intervening when necessary [1].

The concept of a “healing environment” dates back to the Crimean War and the work of Florence Nightingale, who needed little convincing as to the benefits of light, fresh air, and access to nature on recovery from illness and trauma [2]. To make the neonatal intensive care unit (NICU) a place where premature and sick full-term babies can heal, it is important to pay close attention to the temperature, touch, postural control, smell, taste, sound, and light, as well as the space, privacy, and safety [3].

Clustered care represents clustering nursing care procedures, and several routine practices performed together instead of spacing them out over time. The main goal of clustering care is to allow the premature infant to rest for longer periods [4, 5].

Newborn infants admitted to the NICU are constantly handled, which disturbs their sleep patterns. Therefore, to ensure that infants get enough undisturbed sleep, daily routines like cleaning, changing diapers, oil massage, weighing, putting caps, gloves, and socks, giving antibiotics, and feeding can be easily done by the nurse together in a cluster when the infant is awake instead of disturbing the sleep by doing these activities at different times [6].

The NICU is considered a harsh and developmentally unexpected environment, where even basic care can be stressful and unpleasant for premature infants, being especially essential to reduce their stress and pain. The physiological cues that indicate pain are changes in heart rate, respiratory rate, color, tremors, and twitching. Visceral signs like a hiccup, coughing, spitting, drooling, and vomiting are also cues that the infants need to be calmed before the activity is continued [7]. Consequently, our study aimed to assess the effects of a healing environment and clustering nursing care on premature infants' vital signs, pain, and sleeping.

### Research hypothesis

H_1_: Creating a healing environment and clustering nursing care will improve premature infants' vital signs, pain, and sleeping.

## MATERIAL AND METHODS

An experimental research design was conducted from January 2021 to the end of July 2021. The study was carried out in the neonatal intensive care units (NICUs) at Children's Hospital and Maternity & Gynecological Hospital affiliated with Ain Shams University. A purposive sample was composed of study and control groups. The first 59 premature infants newly admitted to the NICU “control group” were randomly assigned to the previously mentioned setting, but four premature infants were excluded due to complications, and two were discharged. Following the control group, the next 60 premature infants newly admitted to the NICU were assigned to the “study group”, which excluded three premature infants due to complications and four premature infants due to discharge.

Inclusion criteria

- Gestational age between 30 and <37 weeks;
- Birth weight: 1000–2000 g.

Exclusion criteria

- Premature infants with major health issues (such as serious infectious diseases and necrotizing enterocolitis), a congenital anomaly, or hemorrhagic/ischemic brain injury, as well as those who required surgical intervention;
- Premature infants on mechanical ventilation, phototherapy, and sedative medicines.

### Sample size

The sample size was estimated based on the findings of Faul et al. [8] and Liaw et al. [9]. The effect size was 0.51, based on the mean PIPP scores (9.52 and 6.39) and SDs (4.95 and 3.35) during heel stick for routine care and non-nutritive sucking treatments, respectively, and statistical power of 90%, level of confidence was 95%, with Alpha 0.05, Beta 0.1. The sample size, which was established at 53 premature infants, was determined by each group. After accounting for 10% sample attrition (5–6 newborns), each group's ultimate sample size was 59 preterm babies. A test comparing two means was used to determine the sample size using a sample size calculator [10].

### Tools

*Part I: Neonatal characteristics* such as gestational age, Apgar score, sex, weight, length, diagnosis, head circumference, and type of delivery.

*Part II: Vital Signs Assessment Sheet*. It was used to assess the premature infant's vital signs, including respiratory rate, blood pressure, pulse, and oxygen saturation.

*Part III: Premature Infant Pain Profile* was adopted from Stevens et al. [11]. It consisted of seven pain measurement indicators: gestational age, behavioral state (BS) and heart rate, O^2^ saturation, brow bulge, eye squeeze, and nasolabial furrow. A scoring of up to four points (0,1,2,3) was used for each of the seven indicators of PIPP. If the sub-total score was >0, we added the gestational age and BS indicators. The total score was calculated as follows: sub-total score + GA score + BS score. A mean score of <5 represents mild pain, 5–10 moderate pain, and >10 severe pain.

*Part IV: Neonatal behavioral state* was adopted from Saliba et al. [12]. It was used to assess the behavioral state of premature infants post-feeding in both groups by assessing the number of minutes for these states: quiet sleep, active sleep, quiet awake, active alert, and crying.

### Reliability

Reliability was measured through Cronbach's alpha test. The reliability of the Vital Signs Assessment Sheet was 0.768, which means acceptable reliability. Premature Infant Pain Profile reliability was 0.877, which means good reliability. Finally, the neonatal behavioral state reliability was 0.779, which means acceptable reliability.

### Fieldwork

#### Control group

The nurse routinely performed the care for them, and then the researcher examined the sleep behaviors using the neonatal behavioral states, ranging from quiet sleep to full crying, and the vital signs using the vital signs assessment sheet for 45 minutes after the premature infant was fed. No intervention was performed on the premature infants during the 45 minutes of sleep behavior observation. The premature infant pain profile was recorded half an hour after the routine daily care, which involved changing the diaper, eye care, cord care, and feeding at 10.00 a.m. and 1 p.m.

#### Study group

After ensuring that the nurses mastered the skills and knowledge to create a healing environment and clustering nursing care, they applied it for an entire week by:

*Controlling light* through adjustable lighting at each incubator, using the minimal amount of light needed to accurately assess and manage premature infants, protecting their eyes from direct light during assessments, and using heavy incubator covers to limit excessive light exposure during sleep.

*Controlling noise* by speaking as quietly as possible, avoiding loud laughter or conversations, avoiding talking beside the incubator or allowing medical rounds too close to the bedside, in addition to a quiet alarm as quickly as possible, using blankets or covers over the incubator to attempt to muffle the noise, avoid placing items like files, stethoscopes cups, and other equipment on the incubator, close incubator portholes, drawers and refrigerator gently, use cell phones on vibrate mode rather than audible alarm mode.

*Smell:* Maintaining a scent-free unit for staff, and encourage parents to limit the use of fragrance, avoid the odor of smoke on caregivers' clothing, provide mother's scent, when possible, with a breast pad or soft cloth, and offer pacifier dips with mother's milk.

*Touch:* Facilitate skin-to-skin opportunities, utilize supportive positioning devices such as the DandleRoos nest or wraps and change the infant's position slowly and without sudden movements. Encourage frequent and extended periods of skin-to-skin care. Demonstrate gentle but firm static containment (quiet hands or hand hugs).

*Clustering nursing care:* Identify five nursing procedures performed without interruption, such as eye care, diaper care, weighing, replacing the pulse oximetry probe, and one of the invasive interventions performed by a trained nurse, such as blood vessel replacement, suctioning, or blood sampling.

After creating the healing environment and clustering nursing care for one week, the researcher examined the sleep behaviors using the neonatal behavioral states, which range from quiet sleep to full crying, and the vital signs using the vital signs assessment sheet for 45 minutes after the premature infant was fed. No intervention was performed on the premature infants during the 45 minutes of sleep behavior observation. The premature infant pain profile was recorded half an hour after the routine daily care, which involved changing the diaper, eye care, cord care, and feeding at 10.00 a.m. and 1 p.m.

### Statistical analysis

The collected data were coded and entered into the statistical package for social sciences (SPSS) (SPSS Inc; version 24; IBM Corp., Armonk, NY, USA). The Chi-square test was used to analyze categorical variables. A t-test was used to compare the means between the two groups. The results were considered statistically significant at P<0.05 and highly significant at P<0.01**.

## RESULTS

More than one-third (39.6% and 37.7%) of premature infants in both groups had a gestational age between 32 and <34 weeks (Table 1). There were 58.5% girls in the study group and 64.2% in the control group. The mean birth weight in the study group was 1.340±125.3 and 1.366±170.12 in the control group. Furthermore, the mean head circumference was 30.24±1.88 in the intervention group and 30.39±1.7 in the control group. The mean Apgar score at 5 minutes was 9.04±0.45 and 9.20±0.64 for the study and control groups, respectively.

**Table 1:** Distribution of premature infants according to their characteristics in study and control groups (n=53).

Premature characteristics	Study group		Control group		Test
	N=53	%	N=53	%	P-value
**Gestational age (weeks)**					T-test
30–32	17	32.1	19	35.8	1.890 >0.05
32–34	21	39.6	20	37.7	
34–36	15	28.3	14	26.5	
Mean±SD	32.17±1.20		31.99±1.10		
**Sex**					Chi-square
Boy	22	41.5	19	35.8	2.976
Girl	31	58.5	34	64.2	>0.05
**Birth weight**					1.253
Mean±SD	1.340±125.3		1.366±170.12		>0.05
**Length**					1.807
Mean±SD	43.70±2.80		43.21±1.99		>0.05
**Head circumference**					1.205
Mean±SD	30.24±1.88		30.39±1.76		>0.05
**Type of delivery**					Chi-square
Vaginal	40	75.5	36	67.9	3.998
Cesarean section	13	24.5	17	32.1	<0.05*
**Apgar score at**					
1^st^ minute	8.1±0.97		8.34±0.78		>0.05
5^th^ minute	9.04±0.45		9.20±0.64		>0.05

* – Significant at p<0.05. ** – Highly significant at p<0.01. Not significant at p>0.05.

There were highly significant differences between the study and control groups regarding the mean time of the various stages of sleep and awake state (p-value<0.01**) (Table 2).

**Table 2:** The mean time of sleep and wake state in the premature infant in study and control groups (n=53).

Items	Study	Control	T-test	P-value
	Mean±SD	Mean±SD		
Quiet sleep	19.33±4.78	6.42±2.87	19.645	<0.01**
Active sleep	24.66±4.71	21.90±3.99	11.076	<0.01**
Quite awake	2.11±0.76	5.16±1.12	12.532	<0.01**
Active alert	3.11±1.02	9.57±3.11	10.138	<0.01**
Crying	0.87±0.08	1.95±0.68	8.102	<0.01**

* – Significant at p<0.05. ** – Highly significant at p<0.01. Not significant at p>0.05.

Table 3 revealed highly statistically significant differences between the study and control groups concerning respiration (p-value<0.01), heart rate, systolic blood pressure, and O^2^ saturation (p-value<0.05). However, there were no statistically significant differences regarding temperature and diastolic blood pressure between the study and control groups.

**Table 3:** Mean score levels of vital signs among premature infants in study and control groups (n=53).

Vital signs	Study	Control	T-test	P-value
	Mean±SD	Mean±SD		
Respiration	41.13±4.70	47.60±5.78	8.712	<0.01**
Heart rate	134.1±13.8	138.02±15.6	4.076	<0.05*
Temperature	36.82±0.31	36.91±0.42	1.098	>0.05
Systolic blood pressure	58.63±7.58	62.80±8.01	3.991	<0.05*
Diastolic blood pressure	32.46±3.40	33.10±2.99	1.643	>0.05
O_2_ saturation	96.44±2.70	94.29±3.29	4.865	<0.05*

* – Significant at p<0.05. ** – Highly significant at p<0.01. Not significant at p>0.05.

Table 4 illustrated highly significant differences in the mean scores of changes in HR between the study and control groups (p-value<0.01**) and significant differences regarding other items from the pain profile scale at p-value<0.05*.

**Table 4:** The mean score levels of pain profile among premature infants in study and control groups.

Items	Study	Control	T-test	P-value
	Mean±SD	Mean±SD		
Change in HR	3.23±0.25	9.34±4.80	8.368	<0.01**
Decrease O_2_ saturation	2.11±0.65	4.70±1.99	5.142	<0.05*
Brow bulge (sec)	2.90±0.35	5.60±1.46	5.973	<0.05*
Eye squeeze (sec)	2.45±0.76	6.11±2.07	4.706	<0.05*
Nasolabial furrow (sec)	2.70±0.59	7.08±0.68	6.111	<0.05*
GA	32.17±1.20	31.99±1.10	1.890	>0.05

* – Significant at p<0.05. ** – Highly significant at p<0.01. Not significant at p>0.05.

**Figure 1:**
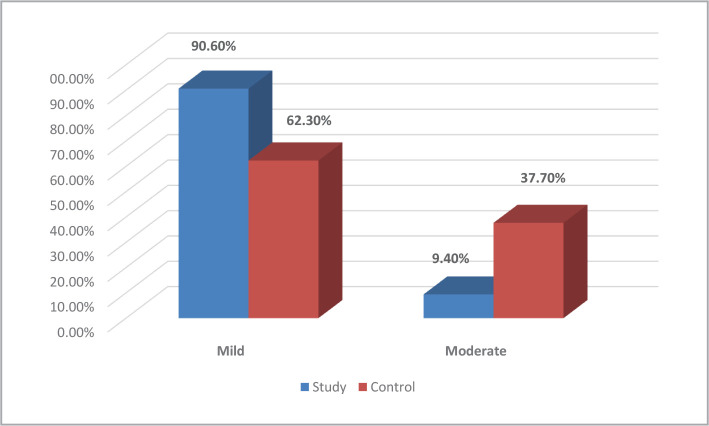
Distribution of premature infants according to their total pain profile in study and control groups (n=53). X^2^ 8.914; p-value<0.01**.

The majority of premature infants (90.6%) in the study group had mild total premature infant pain profiles. In comparison, more than one-third (37.7%) in the control group had moderate total premature infant pain profile scores, with a highly statistically significant difference between the groups (p-value<0.01) (Figure 1).

## DISCUSSION

Premature infants' brains and sensory systems are affected by NICU stimulation. To create a healing environment, background neurosensory stimulation must allow sensory systems to distinguish and accommodate significant signals [13].

Researchers were careful to choose a sample that fit the planned criteria, and the current results showed that the research and control groups were similar in length, head circumference, birth weight, gestational age, medical diagnosis, and Apgar score (p>0.05).

Regarding the vital signs of premature infants, there were highly significant differences between the study and control groups concerning respiration (p-value<0.01), heart rate, systolic blood pressure, and O^2^ saturation (p-value<0.05). These results correspond with the study performed by Başaranoğlu et al. [14] who observed significant differences in heart rate and oxygen saturation when newborns were exposed to noise. The physiological signs of premature infants improved after applying clustering nursing care, therefore, this type of care is recommended for premature babies [15].

According to sleep and wake time, our study revealed a statistically significant difference in the mean score levels of organization state/sleep & responsiveness/interaction between the groups (p-value<0.05). On the other hand, there was no statistically significant difference between motor & autonomic domains (p-value>0.05). These results are supported by the clinical trial conducted on 60 neonates by Bazregari et al. [16], who pointed out that cluster care can significantly boost neonates' calm and active sleep. The results suggest including this type of care in the NICU program, student curricula, and nursing retraining. Nurses are crucial for preterm infant sleep therapies [17].

Finally, regarding pain profile among premature infants, there was a highly significant difference in the mean score of change in HR (p-value<0.01) between the groups. Furthermore, we identified significant differences between the study and control groups regarding other items from the pain profile scale (p-value<0.05).

These results agreed with Chuang et al. [18], who concluded that bundled developmental care lowered newborns' pain, stress, and recovery time. Moreover, Sizun et al. [19] reported that developmental care reduced pain and hypoxia episodes after typical nursing interventions.

## CONCLUSION

Applying a healing environment and clustering nursing care significantly improved respiration, heart rate, oxygen saturation, and systolic blood pressure. Furthermore, it increased the time of sleep and decreased the waking state in the study group. There were statistically significant differences between the study and control groups regarding the items from the pain profile scale. Most premature infants in the study group had mild pain scores, while more than one-third in the control group had moderate total pain profile scores.
